# EC2Seq2Sql: Patient-trial matching with LLM agents

**DOI:** 10.1371/journal.pone.0341827

**Published:** 2026-02-12

**Authors:** Liu Yang, Yongzhong Han, Liang Liu, Xiaoyan Jiang, Ying Li, Jihan Huang, Qianmin Su

**Affiliations:** 1 School of Electronic and Electrical Engineering, Shanghai University of Engineering Science, Shanghai, China; 2 Clinical Research Unit, Institute of Clinical Science, Zhongshan Hospital, Fudan University, Shanghai, China; 3 Leiden Institute of Advanced Computer Science, Leiden University, Leiden, Netherlands; 4 Department of Hepatology Longhua Hospital, Shanghai University of Traditional Chinese Medicine, Shanghai, China; 5 Center for Drug Clinical Research, Shanghai University of Traditional Chinese Medicine, Shanghai, China; Philadelphia University, JORDAN

## Abstract

Timely identification of patients who meet clinical trial eligibility criteria is a persistent bottleneck in trial recruitment because the criteria are written in flexible natural language, while hospital EHRs are stored in structured schemas. To bridge this gap, we propose EC2Seq2Sql, an end-to-end, two-stage framework that automatically converts narrative eligibility criteria into executable SQL queries for EHR-based patient screening. In the first stage, a BART-based semantic parser transforms free-text trial criteria into lightweight structured pattern sequences defined over seven common clinical domains. In the second stage, an LLM-based agent, guided by system- and human-designed prompts, grounds these structured patterns to the target database schema and generates syntactically valid and logically coherent SQL statements. We evaluated the framework on the ClinicalTrials.gov eligibility-criteria dataset and further validated it on a de-identified real-world hepatocellular carcinoma EHR cohort from Zhongshan Hospital, Fudan University. The BART parser outperformed representative Seq2Seq baselines, achieving ROUGE_L 0.8067 and BLEU 0.8427, while the SQL generation stage reached an exact-match accuracy of 0.84 and an execution accuracy of 0.91 after SQL normalization. On the real-world cohort, the generated queries achieved a clinical match accuracy of 0.88 after expert review, indicating that the proposed pipeline can retrieve trial-eligible patients from operational EHR data. These results suggest that EC2Seq2Sql can substantially reduce manual screening effort and provide a reproducible path from narrative criteria to database-level cohort identification, although broader multi-center validation and ontology-based normalization will be needed for large-scale deployment.

## Introduction

Clinical trials are a cornerstone of modern medical research, providing the primary evidence for evaluating the safety and efficacy of new drugs, treatments, and medical devices [1]. Despite their scientific value, many trials are delayed or under-enrolled due to patient recruitment bottlenecks: more than half of trials report recruitment-related delays, one-third of publicly funded trials require extended timelines, and up to one quarter of cancer trials fail to reach their target sample size [2,3]. Manual screening of Electronic Health Records (EHRs) against trial criteria is time-consuming, labor-intensive, and susceptible to subjective bias, and recruitment costs can account for over 30% of the total trial budget [4,5]. Therefore, there is a clear need for automated, accurate, and scalable patient-screening strategies to improve clinical trial efficiency.

Clinical trial eligibility criteria (EC) define the inclusion and exclusion conditions under which a patient can participate in a study. EC are usually written in free-form natural language by domain experts to ensure scientific rigor, ethical compliance, and data validity [6,7]. In parallel, the widespread adoption of EHR systems has created large, longitudinal, and multimodal clinical data repositories that theoretically make automated trial matching feasible. However, a substantial gap remains between narrative EC and structured EHR data [8]. EC often contain domain-specific expressions, temporal constraints, and composite logical conditions, whereas EHR databases organize diagnoses, procedures, laboratory tests, and medications into heterogeneous, sometimes institution-specific schemas. As a result, accurately identifying patients who satisfy the EC in real time remains a non-trivial task. In parallel with these developments, complementary lines of research have highlighted the value of richer patient representations and attention-based deep models in clinical applications. Alanazi et al. integrated wearable sensor streams with EHR data to construct more comprehensive longitudinal patient profiles for downstream decision support, demonstrating how multimodal digital traces can enhance the quality of electronic health records [9]. In the imaging domain, graph attention networks and enhanced U-Net variants with multi-scale attention have achieved substantial gains in lesion segmentation and classification, further illustrating the effectiveness of attention mechanisms for modeling complex clinical signals [10,11].

To close this gap, an automated pipeline must (1) interpret natural-language EC with sufficient semantic fidelity, (2) normalize them into a structured representation that is compatible with common clinical domains, and (3) generate executable database queries over EHR tables. Existing rule-based or template-based approaches can support fixed sentence patterns, but they are difficult to generalize to complex or evolving EC and require substantial manual engineering [5,12]. Recent deep learning advances suggest that transformer-based models are well suited for capturing long-range dependencies and clinical semantics, but they still need to be grounded to the actual database schema to be clinically useful.

In this context, we propose EC2Seq2Sql, an end-to-end framework that integrates the semantic parsing capability of the Bidirectional and Auto-Regressive Transformer (BART) model with an LLM-based agent to automatically transform narrative EC into SQL queries executable on EHR databases. First, EC2Seq2Sql uses BART to perform semantic parsing and converts unstructured EC text into lightweight, structured pattern sequences. These patterns are defined over seven frequently used clinical domains—condition, procedure, observation, laboratory test, drug, gender, and age—so that most common trial constraints can be captured in a uniform manner. Second, the structured patterns are passed to an agent that is guided by system- and human-designed prompts to generate syntactically correct and logically coherent SQL statements. Finally, the generated SQL queries can be run directly on the target EHR database to retrieve candidate patients who meet the trial conditions. By automating the full EC-to-SQL pipeline, the proposed method reduces manual workload, improves screening consistency, and provides a technical foundation for intelligent clinical trial recruitment.

## Related work

In early studies, rule-based and template-matching techniques were commonly employed to directly translate keywords and logical statements from natural language into SQL queries. The EliXR model [13] primarily focuses on the semantic parsing and logical representation of clinical trial eligibility criteria. The EliXR-TIME model [14] employs a rule-based approach to convert time-related eligibility criteria into SQL queries, enabling efficient querying within a database. This approach is effective when the sentence structure is fixed; however, its applicability and performance diminish when the eligibility criteria involve complex semantic expressions or variable structures.

The i2b2 platform [15,16] enables researchers to specify conditions (e.g., diagnoses, medications, laboratory results, demographic information, etc.), with the platform dynamically generating SQL queries to identify patients who meet the eligibility criteria within the EHR database. Although i2b2 offers a modular graphical interface, it still necessitates significant user involvement and logical understanding during query construction, which can pose a barrier, particularly for users without a technical background. To facilitate query definition and execution for non-technical researchers, Leaf [17] provides a user-friendly interface that dynamically transforms query parameters into SQL statements through interactive actions. However, both platforms offer inadequate fine-grained support for handling complex temporal logic and advanced constraints. In contrast, advanced NLP-based approaches demonstrate greater power and flexibility.

Criteria2Query [18] is a hybrid information-extraction framework integrating machine learning with rule-based methods. It maps free-text eligibility criteria to a standardized semantic model and generates SQL queries compatible with clinical databases such as the OMOP Common Data Model (CDM) [19]. Subsequent versions, including Criteria2Query 2.0 [20], incorporated statistical learning and human–machine collaboration, allowing users to refine generated SQL queries. Recent advancements in large language models (LLMs) have led to Criteria2Query 3.0 [21], which leverages GPT-4 for concept extraction, SQL generation, and reasoning. This approach semi-automatically converts free-text eligibility criteria into executable clinical database queries. Recent work has also started to systematically validate LLM-generated, executable queries against standardized clinical data models. For example, Lee et al. [22] evaluated LLM-based conversion of free-text eligibility criteria into OMOP CDM–compatible SQL and reported common failure modes such as hallucinated concepts and domain misassignment, highlighting the need for careful grounding and validation in real-world deployments.

Beyond Criteria2Query, encoder–decoder neural architectures have been applied to Text-to-SQL tasks [23–27]. For example, the sequence-to-sequence (Seq2Seq) model [25] employs a BiLSTM encoder to capture semantic features and an attention-based decoder to generate SQL statements, while Pan et al. [26] adopted BERT–Transformer hybrids for contextual understanding. More recently, agentic LLM-based Text-to-SQL frameworks have incorporated question routing, schema selection, and explicit syntax/execution verification to improve execution accuracy [28].

In parallel with these neural Text-to-SQL studies, the rapid evolution of LLMs has further advanced the automation of patient–trial matching and eligibility criteria interpretation. A recent scoping review further summarized emerging LLM applications for patient–trial matching, and emphasized persistent challenges in generalizability, interpretability, and grounding to real-world clinical data [29]. Jin et al. [30] introduced an LLM-driven framework in which patient profiles and trial eligibility texts are jointly represented and ranked to retrieve candidate trials and perform (near) zero-shot patient–trial matching. While effective in semantic retrieval, it lacks structured query generation and explicit linkage to database schemas, limiting interpretability and reproducibility on real EHRs. Lee et al. [31] proposed CriteriaMapper, which normalizes eligibility criteria and patient features via rule-based and embedding-assisted alignment. Although it improves terminology consistency, it depends on deterministic rules and does not support compositional SQL generation or execution-level evaluation. Ferber et al. [32] developed an end-to-end GPT-style pipeline for cohort identification across synthetic EHR datasets; while demonstrating scalability, it omits grounding to real hospital databases and metrics such as execution accuracy.

As summarized in Table 1, existing systems differ significantly in their methodological foundations and levels of automation. Rule-based and interface-driven tools provide limited support for complex logic and require substantial user involvement, while recent LLM-based approaches emphasize semantic matching but generally lack executable query generation and validation on real-world EHR databases. These recent studies collectively underscore a growing trend toward leveraging LLMs for clinical trial recruitment automation. Building upon this direction, the proposed EC2Seq2Sql framework advances the field by introducing a two-stage architecture that combines transformer-based semantic parsing (BART) with an LLM-driven agent for executable SQL generation. Unlike prior end-to-end models, EC2Seq2Sql explicitly bridges natural-language criteria and structured database queries, enabling interpretability, full executability, and real-world validation on hospital EHR data.

**Table 1:** Comparison of representative systems for eligibility criteria interpretation and patient–trial matching.

Study	Approach Type	Core Functionality	Automation Level	Limitations
EliXR/EliXR-TIME [13,14]	Rule-based semantic parsing	Template-based EC interpretation	Low	Limited generalization; struggles with complex or variable expressions
i2b2 [15]	Graphical interface + rule logic	User-driven construction of logical patient cohort queries	Medium	Requires clinical reasoning by user; weak temporal logic support
Leaf [17]	Interactive query interface	Dynamic parameter-driven EHR search through UI actions	Medium	Lacks automated semantic parsing; insufficient for complex eligibility constraints
Criteria2Query [18,20,21]	Hybrid IE + GPT-4 assisted reasoning	EC normalization and OMOP-compatible SQL generation	Medium	Manual refinement often required; execution correctness not guaranteed
Text-to-SQL [25,28]	Encoder–decoder neural architectures	Direct natural language to SQL generation	Medium	Schema-sensitive; logical consistency and SQL correctness remain challenging
TrialGPT [30]	End-to-end LLM semantic matching	Zero-shot patient–trial retrieval and ranking	High	No SQL grounding; results cannot be verified directly against EHR databases
CriteriaMapper [31]	Rule + embedding-based concept normalization	EC–patient semantic alignment for cohort identification	Medium	No compositional reasoning or SQL synthesis
LLM-based Matching [32]	End-to-end GPT-style inference pipeline	Matching performed on synthetic EHR datasets	High	Not evaluated on real hospital data
**EC2Seq2Sql(This Work)**	**BART parser + LLM SQL agent**	**Two-stage EC → structured representation → executable SQL → real EHR retrieval**	**High**	**Ontology alignment needed for multi-institution generalization**

## Materials and methods

### Overview of the method

**Fig 1:**
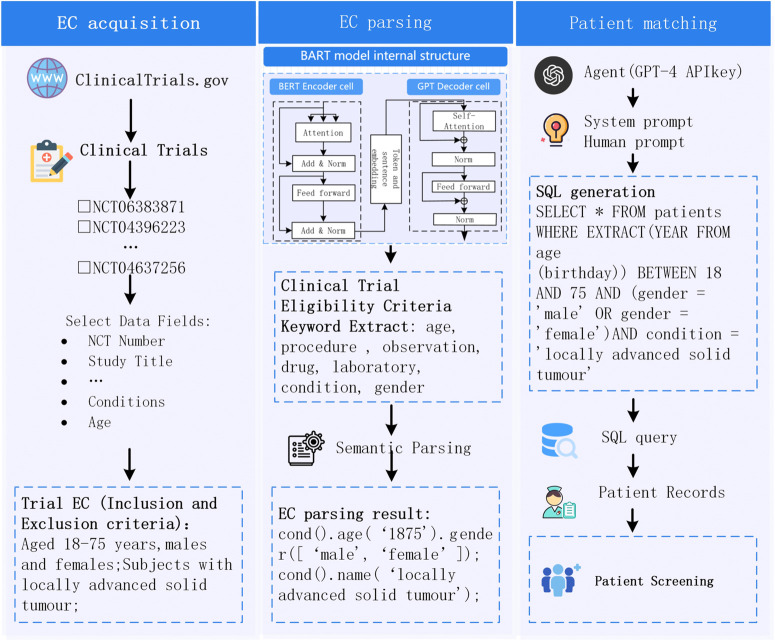
The overall framework of EC2Seq2Sql. The workflow starts from clinical trial eligibility criteria collected from ClinicalTrials.gov (input), then parses the free-text criteria into lightweight structured patterns using a BART-based semantic parser, and finally converts the structured patterns into executable SQL through an LLM-based agent to retrieve eligible patients from the EHR database (output). The three main stages are (1) eligibility criteria acquisition, (2) eligibility criteria parsing, and (3) patient matching, which are connected by directional arrows to indicate the processing order. Each stage produces intermediate outputs (retrieved trials, structured patterns, SQL queries) that serve as inputs to the subsequent stage.

To automate the conversion of clinical trial eligibility criteria from natural language into SQL queries, the EC2Seq2Sql method proposed in this study integrates BART with LLM agents. Fig 1 illustrates the overall framework, which is composed of three main components: eligibility criteria acquisition, eligibility criteria parsing, and patient matching. The specific process is summarized as follows.

(1) Eligibility Criteria Acquisition: The researcher identifies relevant clinical trials by searching for keywords in the clinical trial protocol using the ClinicalTrials.gov platform. Subsequently, the researcher screens and selects trials that meet the specified criteria based on the retrieved information.

(2) Eligibility Criteria Parsing: Eligibility criteria are semantically parsed using the BART model to generate lightweight and structured information.

(3) Patient Matching: Lightweight and structured information is converted into SQL query statements through the use of agents, integrating both human- and system-prompted engineering. The SQL query is then executed on the EHR database to identify patients who meet the eligibility criteria.

### Eligibility criteria acquisition

ClinicalTrials.gov is a comprehensive global database for clinical trials, maintained and operated by the U.S. National Library of Medicine and the National Institutes of Health. Launched in 2000, the platform houses clinical trial data from a global network of studies. The platform offers comprehensive information, including fundamental details of clinical trials, as well as insights into trial design and methodology. Researchers can utilize the platform to identify and select relevant clinical trials and extract both inclusion and exclusion criteria from the selected trials.

### Eligibility criteria parsing

As the eligibility criteria retrieved from the ClinicalTrials.gov platform are presented in natural language text, the critical step in accurately parsing and generating SQL queries involves converting these textual descriptions of clinical trial eligibility into lightweight and structured patterns. This process requires not only the accurate extraction of key entities from the text, but also a thorough understanding of the logical relationships between the eligibility criteria to generate valid SQL query statements. To accomplish this objective, this study employs the BART model [33], which leverages its encoder to capture the semantic features of the input text. By utilizing its robust bidirectional contextual understanding and generative capabilities, BART is employed to parse complex natural language texts. Subsequently, the encoder of the BART model extracts semantic information from the input text using the self-attention mechanism. For instance, in the phrase “patients aged 18 to 65 and diagnosed with type 2 diabetes,” BART effectively extracts the key information, such as ‘18 to 65 years old’ and ‘type 2 diabetes,’ while also comprehending the logical relationship between these elements. Through its multi-head self-attention mechanism, BART is capable of focusing on critical information within the text, particularly when handling complex semantics and logical relationships, such as combinations of multiple conditions, thereby effectively capturing the dependencies between these conditions. This enables BART to accurately interpret and parse clinical trial eligibility criteria that involve multiple conditions.

Since the parsed results often involve complex logical structures and nested expressions, such as logical operators, entity-attribute mappings, and temporal conditions, the system must transform the extracted entities into lightweight and structured patterns. The objective of the parsing process is to generate a set of lightweight and structured patterns that can be processed by the agent. These patterns are highly scalable and capable of adapting to a wide range of eligibility criteria, including complex combinations of conditions. Ultimately, by leveraging the BART model for natural language text parsing, complex clinical trial eligibility criteria are transformed into lightweight and structured patterns. This process enhances both the efficiency and accuracy of parsing while also establishing a robust foundation for the subsequent generation of SQL queries. The design of the lightweight and structured model equips the system with extensive adaptability and scalability, enabling it to accommodate the diverse requirements of clinical trials and efficiently manage complex combinations of various trial conditions.

Considering the broad scope and complexity inherent in formulating eligibility criteria, this study synthesizes relevant literature and expert insights to define seven domains that encompass the conditions of most clinical trial eligibility criteria [7]. The identified fields are as follows: condition, procedure, observation, laboratory, drug, age, and gender. Detailed descriptions of these fields are provided in Table 2.

**Table 2:** Definitions of the seven clinical domains used in this study.

Name	Meaning
condition	This refers to specific health conditions, diseases, symptoms, or medical states, such as diabetes, HIV infection, and retinopathy. The label is applied to text that describes specific illnesses or health statuses.
procedure	This refers to a sequence of actions performed in a specific order or manner, such as surgical procedures or diagnostic tests.
observation	Refers to the process of monitoring or recording a fact, event, or condition for medical purposes.
laboratory	Refers to laboratory test results, such as blood tests, kidney function assessments, metabolic indicators, liver function tests, and other related evaluations.
drug	Denotes medications or pharmaceutical interventions employed to treat or manage diseases and symptoms.
age	Represents the age range of patients included in the inclusion/exclusion criteria for clinical trials.
gender	Refers to the gender of patients (male, female, or other) as defined in the eligibility criteria for clinical trials.

### Patient matching

**Fig 2:**
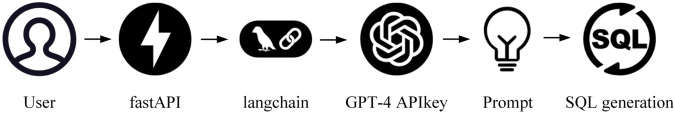
SQL generation process. This figure details the workflow that transforms the structured eligibility patterns into an executable SQL query. The input is the lightweight, seven-domain structured representation produced in the previous stage. First, the front-end sends the structured input to the FastAPI service, which forwards it to the LangChain-based workflow. LangChain constructs a prompt that combines a system prompt (task description and database context) and a human prompt (explicit inclusion/exclusion conditions). Guided by this hierarchical prompt, the GPT-4 model generates a syntactically correct and schema-aligned SQL statement. The final output is an SQL query that can be executed on the hospital EHR database to return the list of patients matching the criteria.

Generative artificial intelligence powered by foundation models supports the development and deployment of agents that utilize advanced reasoning and language processing capabilities, allowing them to play a proactive and autonomous role in achieving users’ goals. To identify patients who fulfill the clinical trial eligibility criteria, LLM agents must convert the lightweight and structured information generated by the BART model into efficient SQL queries. The process of SQL generation is illustrated in Fig 2. First, the user enters the lightweight and structured information generated by the BART model via the front-end interface. This data is then received and preprocessed by FastAPI. Next, the LangChain framework processes the user’s input and constructs a request tailored for the language model, utilizing predefined prompt strategies. Subsequently, following the prompt’s guidance, the GPT-4 model transforms the natural language input into an SQL query that conforms to both grammatical and logical standards.

The process primarily involves the following aspects:

(1) System and human prompt design

To ensure the efficiency and accuracy of the generated SQL queries, an interactive mechanism between the system and human prompts was developed. The system prompts are designed to supply the model with task objectives and essential information, thereby guiding it in generating SQL queries that fulfill the specified requirements. The content of the system prompt is as follows: “You are an expert in generating SQL queries for clinical trials. Your task is to create SQL queries based on lightweight, structured semantic representations to filter patients who meet specific inclusion and exclusion criteria from EHR databases. Each query should comprehensively address the following seven core fields: condition, procedure, observation, laboratory, drug, age, and gender. It is essential to ensure that the generated SQL query is syntactically correct, logically sound, and accurately reflects the conditions and constraints outlined in the eligibility criteria.” The purpose of the human prompt is to define the conditions that the generated SQL query must satisfy. The content is as follows: “Using the provided lightweight and structured semantic representation, generate an SQL query that parses and incorporates information from the following fields: condition, procedure, observation, laboratory, drug, age, and gender. Additionally, ensure that time expressions and logical operators are correctly converted into syntactically valid database expressions so that the query accurately reflects the time constraints and logical relationships specified in the eligibility criteria.”

(2) LangChain-based workflow

This study introduces a method based on the LangChain framework to automate the patient data screening process and efficiently identify individuals who meet the inclusion and exclusion criteria for clinical trials [34]. This method employs the SQL database class from LangChain to establish a connection to the patient information database, which stores detailed patient records, including age, gender, and medical history. Through interaction with the database, patients who satisfy the clinical trial eligibility criteria can be automatically screened.

The initial step in generating and executing SQL queries involves interacting with the LLM through the PromptTemplate in LangChain. The PromptTemplate is employed to extract key information from unstructured or semi-structured text and integrate it into a predefined SQL query template, thereby generating a complete query statement. Subsequently, using the LLMChain class provided by LangChain, the system can automatically tailor and generate the corresponding SQL query according to the user’s requirements.

To further improve query automation, the workflow can be optimized by leveraging the functionality of the LangChain framework. This functionality integrates various components, including database query tools and the LLM, facilitating the dynamic execution of SQL queries and the processing of results. By effectively utilizing these integrated components, the workflow becomes more efficient across multiple stages, such as query generation, execution, and subsequent result processing.

Specifically, LangChain offers the following key components to support this process:

- LLM Wrappers: This component provides interfaces for connecting to various LLMs, including GPT-4 and those available through Hugging Face.
- Prompt Templates: This feature facilitates the creation of reusable text templates, eliminating the need for hard coding and enabling the dynamic insertion of user inputs or variable values, thereby allowing for more flexible requests to the language model.
- Indexes: This feature enhances retrieval efficiency, ensuring the rapid identification of relevant information from large datasets.
- Chains: This feature allows multiple steps or components to be executed in a predefined logical sequence, thereby creating a coherent workflow for developing complex applications.
- Agents: Serving as a coordinator, it allows the language model to interact with external Application Programming Interfaces (APIs) and services, adapt to environmental changes, and facilitate more intelligent task automation.

## Experiment

### Dataset

This study utilizes two complementary datasets: a public benchmark dataset for training and evaluating the semantic parsing model and a real-world EHR cohort for validating the clinical applicability of the proposed EC2Seq2Sql framework.

**Public benchmark dataset.** We used the ClinicalTrials.gov eligibility criteria dataset released by the BioNLP group at the University of Washington [35]. The dataset was accessed on June 1, 2024. All records are fully de-identified and publicly available. Each sample consists of a natural language eligibility criterion paired with a corresponding structured representation. The natural language criteria describe conditions for subject enrollment, including demographic requirements, diagnosed diseases, clinical history, laboratory thresholds, treatment records, and additional restrictions.

To enable machine-readable semantic modeling, the structured representations summarize each criterion into a lightweight symbolic form organized around seven conceptual domains: condition, procedure, observation, laboratory, drug, age, and gender. These domains correspond directly to commonly queried clinical attributes in EHR systems, allowing downstream conversion to executable SQL. This structured abstraction reduces linguistic variability while preserving logical meaning, making it well-suited for Seq2Seq semantic parsing.

**Fig 3:**
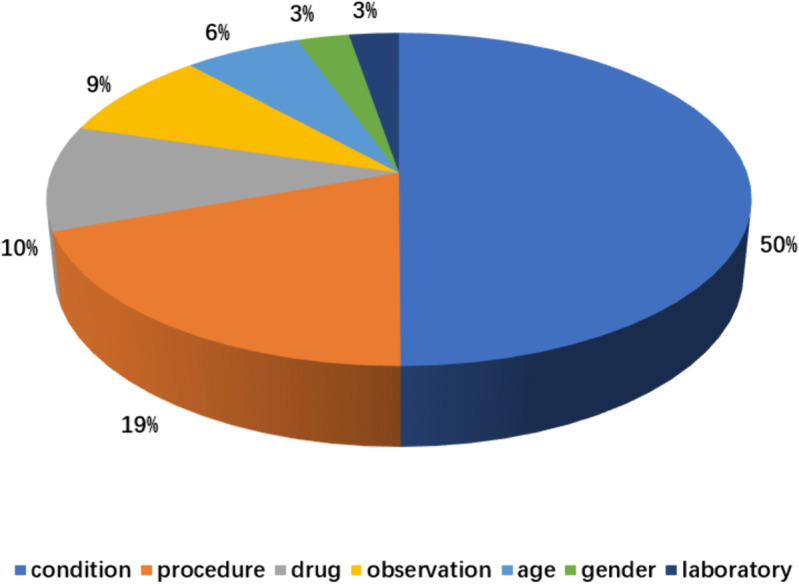
Distribution of the seven conceptual domains. This figure shows how EC elements are mapped to the seven domains (condition, procedure, observation, laboratory, drug, age, gender). It motivates using this schema in the EC2Seq2Sql pipeline.

**Fig 4:**
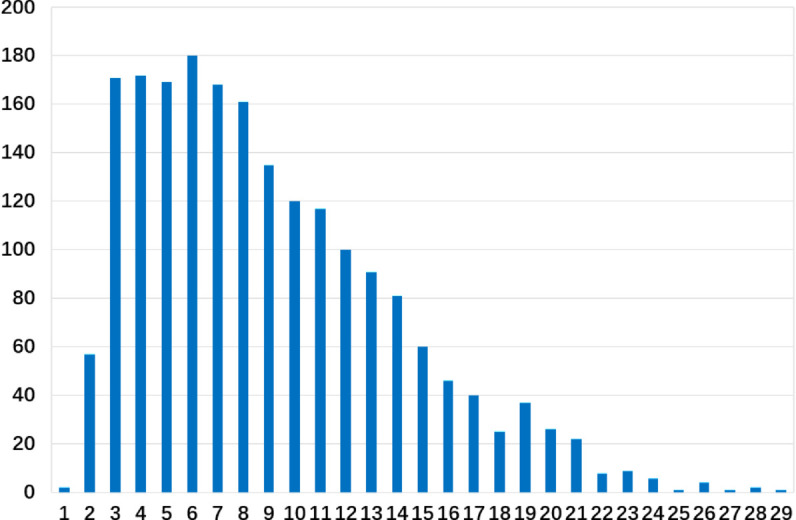
Sentence-length distribution of EC texts. This figure summarizes token lengths of EC sentences in the dataset, indicating the presence of both short rules and long, nested clauses that require transformer-based parsing.

To prevent data leakage, we adopt a trial-aware split, ensuring that criteria from the same clinical trial do not appear across different subsets. The dataset is divided into 80% training, 10% validation, and 10% testing. Fig 3 shows the distribution of the seven conceptual domains. Fig 4 illustrates the sentence length distribution, indicating that the dataset contains both simple rules and complex clauses with nested logical dependencies.

**Real-world clinical validation dataset.** To evaluate the effectiveness of the generated SQL in actual patient-matching scenarios, we further conducted real-world validation on a de-identified hepatocellular carcinoma (HCC) EHR cohort obtained from Zhongshan Hospital. The cohort contains 41 patients diagnosed with HCC, including structured clinical fields such as demographic profiles, serological markers, surgical and systemic treatment histories, immunotherapy usage, and tumor biomarker measurements. All data were de-identified and analyzed under institutional data governance. This dataset enables evaluation of whether the generated SQL queries can accurately retrieve clinically eligible patient subsets, thus demonstrating real-world clinical utility beyond benchmark performance.

Together, these two datasets support both algorithmic evaluation (semantic parsing and SQL correctness) and clinical applicability assessment (patient eligibility retrieval accuracy), fulfilling the complete experimental verification pipeline required for intelligent clinical trial matching.

### Experimental setup

Our experiments are designed to evaluate both the semantic parsing capability and the end-to-end patient matching performance of the proposed EC2Seq2Sql framework. The overall experimental workflow consists of two stages: training a sequence-to-sequence model to convert natural language eligibility criteria into structured semantic representations, and generating executable SQL queries from the structured representations and validating them on real-world patient data.

**Hardware environment.** All experiments were conducted on a high-performance computing platform equipped with an NVIDIA A800 80GB GPU (CUDA 12.2), a 16-core Intel Xeon Platinum 8369C processor, and 255GB of RAM. The computation environment includes a 50GB NVMe data drive to ensure high-throughput data loading during training.

**Semantic parsing model.** We adopt the pre-trained BART-large-CNN model as the backbone encoder-decoder architecture for semantic parsing, which maps natural language eligibility criteria to lightweight structured snippets. The input text is tokenized using the pre-trained BART tokenizer, and the structured representation is linearized as the target output. The model is fine-tuned using the AdamW optimizer with a learning rate of 5e-5, a weight decay of 0.01, a batch size of 4 per device, and 5 training epochs. The best model checkpoint is selected based on validation loss. The hyperparameters are summarized in Table 3.

**Table 3:** BART-large-CNN hyperparameters.

Parameter	Value
Learning Rate	5e-5
Batch Size	4
Number of Epochs	5
Weight Decay Rate	0.01

**SQL generation.** Once the semantic parsing model produces the structured representation, we employ a GPT-4-based reasoning agent to convert the structured form into executable SQL. To ensure deterministic and reproducible SQL outputs, the GPT-4 API is invoked with a temperature of 0, which prevents randomness in generation and guarantees stable comparison across experiments [36]. The agent operates under a hierarchical prompting strategy that enforces correctness in table selection, field grounding, logical operator handling, and numeric range interpretation.

**Evaluation metrics.** We evaluate the semantic parsing stage using Recall-Oriented Understudy for Gisting Evaluation (ROUGE) and Bilingual Evaluation Understudy (BLEU) to measure content-level correctness between the generated and reference structured representations. For the SQL generation stage, we report Exact-set-match Accuracy (EM), which evaluates syntactic alignment with the gold-standard query, and Execution Accuracy (EX), which checks whether the generated SQL returns the correct result set.

For the real-world EHR validation, we further measure Clinical Match Accuracy (CMA), defined as the agreement between model-retrieved patient subsets and expert-reviewed ground truth. This metric reflects the system’s practical utility for clinical trial recruitment.

### Evaluation metrics

#### Evaluation metrics for semantic parsing

In the semantic parsing process, ROUGE and BLEU were employed as the primary evaluation metrics to provide a comprehensive assessment of the model’s performance in the Seq2Seq task. The ROUGE metric comprises ROUGE_1, ROUGE_2, and ROUGE_L, which are employed to evaluate the similarity between the generated text and the reference text. Specifically, ROUGE_1 evaluates word-level matching, ROUGE_2 measures bigram matching, and ROUGE_L assesses structural similarity based on the longest common subsequence. All ROUGE scores are reported as F1 values, which integrate precision and recall to provide a balanced measure of evaluation accuracy and comprehensiveness. Additionally, the BLEU score assesses the quality of the generated text by comparing n-gram overlaps between the generated text and the reference text. The BLEU score not only considers matches from 1-gram to 4-gram but also incorporates a length penalty mechanism to prevent the generated text from being excessively short. Finally, the BLEU score offers a comprehensive assessment of the similarity between the generated text and the reference text. These metrics collectively measure the semantic fidelity and textual similarity between the model’s generated structured representations and the corresponding gold-standard outputs, thereby evaluating the semantic parsing capability of the BART model in transforming natural language eligibility criteria into lightweight structured patterns. The formulas for these metrics are presented as follows:

$$ROUGE\_1=\frac{\sum_{s\in S}{\text{count}}_{1}(s\cap R)}{\sum_{s\in S}{\text{count}}_{1}(s)},$$ (1)

$$ROUGE\_2=\frac{\sum_{s\in S}{\text{count}}_{2}(s\cap R)}{\sum_{s\in S}{\text{count}}_{2}(s)},$$ (2)

$$ROUGE\_L=\frac{\sum_{s\in S}\text{LCS}(s,R)}{\text{len}(s)},$$ (3)

$$BLEU=\min\left(1,\frac{\text{generated\_n\_grams}}{\text{reference\_n\_grams}}\right)\times \exp\left(\sum_{n=1}^{N}p_{n}\right)$$ (4)

In Eq (1), *s* denotes the words in the generated text, *R* represents the words in the reference text, *count*_1_(*s* ∩ R) indicates the number of matching 1-grams between the generated and reference texts, and *count*_1_(*s*) refers to the number of 1-grams in the generated text. In Eq (2), *s* represents the bigrams in the generated text, *R* refers to the bigrams in the reference text, *count*_2_(*s* ∩ R) denotes the number of matching 2-grams between the generated and reference texts, and *count*_2_(*s*) refers to the number of 2-grams in the generated text. In Eq (3), ROUGE_L calculates the Longest Common Subsequence (LCS), where *LCS*(*s*, *R*) represents the length of the longest common subsequence between the generated text *s* and the reference text *R*, and *len*(*s*) denotes the length of the generated text. ROUGE_L emphasizes the sequential consistency between the generated and reference texts, specifically focusing on the alignment between their respective word sequences. In Eq (4), *p*_*n*_ represents the n-gram precision, defined as the ratio of the number of matching n-grams in the generated text to the total number of n-grams in the generated text.

#### Evaluation metrics for SQL generation.

To rigorously evaluate the performance of the proposed EC2Seq2Sql framework in the SQL generation stage, a comprehensive assessment is conducted from two dimensions: syntactic precision and execution correctness. We employ two complementary indicators: EM and EX.

(1) EM

This metric evaluates the syntactic alignment between the predicted SQL query and the gold-standard SQL query. Specifically, it measures whether all SQL clauses and their corresponding tokens exactly match between the predicted query $Q_{pred}$ and the reference query $Q_{gold}$. The indicator is defined in Eq (5):

$$EM=\frac{1}{N}\sum_{i=1}^{N}𝕀(\text{Set}(Q_{pred}^{\left(i\right)})=\text{Set}(Q_{gold}^{\left(i\right)}))$$ (5)

where *N* denotes the total number of test samples, and 𝕀(·) is the indicator function returning 1 if the two token sets are identical and 0 otherwise. As shown in Eq (5), this metric captures the *exact structural correspondence* between the predicted and reference SQL queries, providing a strict measure of syntactic accuracy [37,38].

(2) EX

EX quantifies whether the generated SQL query can be successfully executed and whether its output matches that of the reference query on the same database instance. For each query pair, let $R_{pred}^{\left(i\right)}$ and $R_{gold}^{\left(i\right)}$ denote the result sets returned by executing the predicted and reference SQL queries, respectively. Then, the execution accuracy is computed as Eq (6):

$$EX=\frac{1}{N}\sum_{i=1}^{N}𝕀(R_{pred}^{\left(i\right)}=R_{gold}^{\left(i\right)})$$ (6)

A higher EX score indicates that the model not only generates syntactically valid SQL, but also produces semantically correct query results. As defined in Eq (6), this metric directly reflects the end-to-end usability of the generated SQL [37,38].

### Experimental results

#### Parsed results

In this section, a series of rigorous experiments were conducted within a consistent experimental environment. To ensure a fair comparison, all algorithms were trained and evaluated according to the previously outlined experimental settings. In the semantic parsing process, this study compares a diverse set of models, including traditional Seq2Seq generation models, domain-specific models, and general-purpose LLMs. Specifically, four widely used Seq2Seq models—T5-base, T5-small, GPT-2, and BART-large-CNN—were first evaluated. Among them, T5-base and T5-small are variants of the Transformer architecture [39], with the former having a larger parameter scale and stronger expressive power, while the latter provides higher computational efficiency for lightweight tasks. GPT-2 [40], a general-purpose pre-trained language model, has demonstrated remarkable text generation ability across various natural language processing tasks, and BART-large-CNN, as an encoder–decoder model, is specifically designed for generative applications. To further enrich the comparative analysis, several additional models were incorporated. BioBERT and ClinicalBERT, both pretrained on large-scale biomedical corpora, were introduced to examine the impact of domain-specific pretraining on clinical text understanding [41,42]. GPT-3.5-turbo, a representative LLM, was included to assess the performance difference between fine-tuned encoder–decoder models and general-purpose generative LLMs with strong zero-shot capabilities [43]. In addition, TAPAS, a structured reasoning model designed for table-based natural language understanding, was employed to evaluate the ability of structure-aware models to generate logical and interpretable representations from eligibility criteria [44].

The final evaluation results of all models are presented in Table 4. The experimental results indicate that the BART-large-CNN model outperforms all other models across all evaluation metrics, achieving particularly high scores of 0.8105 in ROUGE_1 and 0.8427 in BLEU, thus demonstrating its exceptional performance in text generation tasks. The T5-base model also demonstrates strong performance, achieving ROUGE_1 and ROUGE_L scores of 0.7277 and 0.7245, respectively, highlighting its competitiveness in text generation. Although the T5-small model offers computational efficiency, its performance slightly lags behind that of the T5-base and BART-large-CNN models, likely due to its smaller number of parameters, which limits its ability to handle more complex text generation tasks. In contrast, the GPT-2 model performed significantly worse than the other models, with ROUGE_1 and ROUGE_L scores of only 0.3223 and 0.2855, respectively, and a BLEU score close to zero (0.0008). This suggests that GPT-2 underperforms on this specific text generation task, likely due to its limitations in processing long texts or generating domain-specific content.

**Table 4:** Performance comparison of different sequence models on the dataset.

Model	ROUGE_1	ROUGE_2	ROUGE_L	BLEU
GPT-2	0.3223	0.0884	0.2855	0.0008
T5-small	0.6069	0.4000	0.5982	0.5925
T5-base	0.7277	0.5482	0.7245	0.7341
BioBERT	0.6724	0.5032	0.6588	0.6781
ClinicalBERT	0.6895	0.5120	0.6703	0.6952
TAPAS	0.7481	0.6103	0.7428	0.7510
GPT-3.5-turbo	0.7836	0.6407	0.7749	0.8013
BART-large-CNN	0.8105	0.6930	0.8067	0.8427

Among the newly introduced models, BioBERT and ClinicalBERT achieved competitive ROUGE and BLEU scores, confirming that biomedical pretraining improves the model’s understanding of clinical trial language and domain-specific concepts. GPT-3.5-turbo demonstrated near–state-of-the-art performance, approaching that of BART-large-CNN without any task-specific fine-tuning, reflecting the strong generalization ability of LLMs on semantic parsing tasks. Meanwhile, TAPAS achieved balanced ROUGE and BLEU scores, indicating its potential for tasks that require logical or structured output generation. Overall, BART-large-CNN remains the top-performing model, reaffirming its effectiveness in producing accurate and coherent structured representations from clinical eligibility texts.

**Fig 5:**
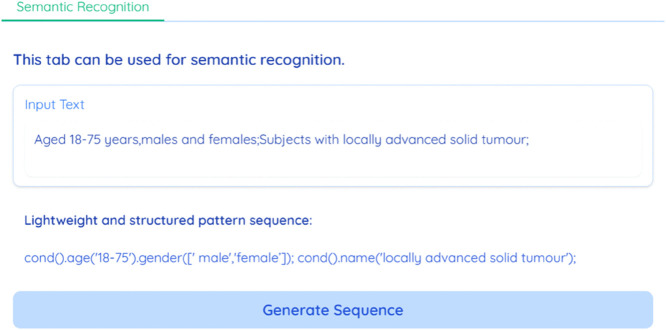
Interface for BART-based EC parsing. Users input free-text eligibility criteria, and the system returns the corresponding lightweight structured pattern, which is then fed to the SQL generation stage.

The system is capable of parsing input text and converting the natural language description of the standard into lightweight and structured data. As illustrated in Fig 5, the input raw text is: “Aged 18-75 years, males and females; Subjects with locally advanced solid tumors confirmed by histopathology.” Upon clicking the “Generate Sequence” button, the system parses the text and generates the structured, lightweight output: “cond().age(‘18-75’).gender([‘male’,‘female’]); cond().name(‘locally advanced solid tumor’).”

#### SQL generation.

To ensure the accurate parsing and conversion of structured patterns into executable database queries, this study employs a dual prompting strategy that integrates both system-level and user-level guidance. The system-level prompt establishes the global context by defining the LLM’s role as an expert in parsing clinical trial eligibility criteria and generating precise SQL statements based on structured pattern inputs. The user-level prompt further specifies the detailed requirements for query construction by emphasizing seven core fields—*condition, procedure, observation, laboratory, drug, age,* and *gender*—thereby ensuring that the generated SQL queries accurately capture the inclusion and exclusion logic of clinical trials.

Guided by these two complementary prompting layers, the agent module receives structured outputs from the BART model and constructs SQL-compliant statements by dynamically integrating system-level and user-level instructions. This hierarchical prompting approach allows the GPT-4-based EC2Seq2Sql framework to maintain both semantic fidelity and structural correctness during query generation.

**Fig 6:**
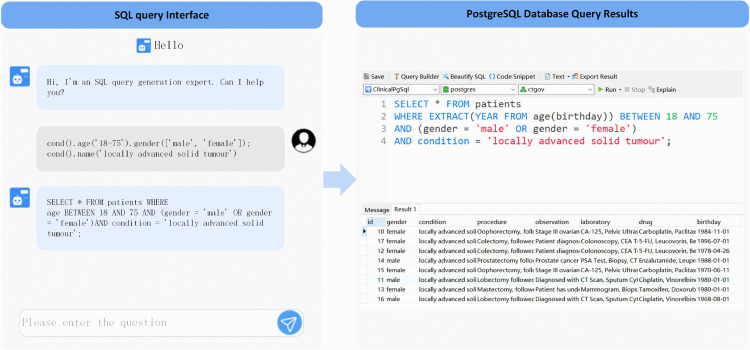
Example of executing an auto-generated SQL for patient retrieval. The SQL produced from the structured eligibility patterns is run on the de-identified hospital EHR to return patients who satisfy the trial criteria; the figure shows this end-to-end result display.

To quantitatively evaluate the reliability and precision of the generated SQL statements, two complementary indicators defined in Section *Evaluation metrics for SQL generation* were employed: EM and EX. Before evaluation, all SQL outputs were normalized for capitalization, clause order, and alias consistency to eliminate non-semantic variations. Experimental evaluation on the benchmark dataset revealed that the proposed GPT-4–based EC2Seq2Sql system achieved an EM of 0.84 and an EX of 0.91, demonstrating high syntactic precision and strong execution-level reliability. These results confirm that the generation pipeline can consistently produce SQL statements that are both executable and semantically faithful to the intended query logic. The SQL generation interface and representative matching results are shown in Fig 6.

### Ablation study

To quantify the contribution of the key components in the proposed EC2Seq2Sql framework, we conducted an ablation study on both the benchmark eligibility criteria dataset and, where applicable, the de-identified hospital EHR cohort. The complete system consists of three major parts: (1) a BART-based semantic parser that converts free-text eligibility criteria into lightweight structured patterns; (2) a seven-domain constraint covering *condition, procedure, observation, laboratory, drug, age*, and *gender*, which enforces clinical completeness; and (3) a GPT-4–driven agent with hierarchical (system + human) prompting that guides schema-grounded SQL generation. We remove each of these parts in turn and re-evaluate the model using the same text-level and SQL-level metrics as in the main experiments. Specifically, ROUGE_L and BLEU are used to assess the quality of the parsed/normalized criteria, whereas EM, EX, and CMA evaluate the executability and clinical matching performance of the generated SQL. The data split and evaluation protocol follow the description in Section *Experimental setup*, including the trial-aware 80/10/10 partition and SQL normalization prior to EM/EX computation.

We examine four variants:

- **(A) Full EC2Seq2Sql**: the complete two-stage pipeline.
- **(B) Without structured patterns**: the agent receives the linearized criteria directly so that we can test whether explicit abstraction into structured patterns is necessary for producing schema-aligned SQL.
- **(C) Without agent prompting**: the structured patterns are preserved, but the hierarchical prompt is replaced by a minimal “generate SQL” instruction in order to measure how sensitive SQL correctness is to prompt design.
- **(D) Without seven-domain constraint**: the parser and the agent prompting are retained, but the explicit requirement of covering all seven clinical domains is removed, simulating scenarios in which only partial attributes are specified.

**Fig 7:**
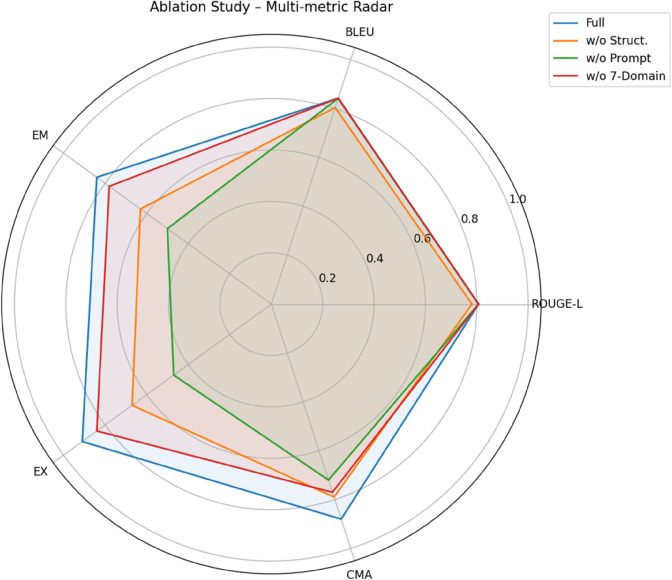
Radar visualization of multi-metric ablation results. The full system encloses the largest area across all five metrics. Removing structured patterns mainly harms EM/EX despite similar text-level scores; removing agent prompting causes the most severe SQL degradation; dropping the seven-domain constraint preserves SQL accuracy but reduces clinical matching on the real-world cohort.

Fig 7 and Table 5 jointly report the results. A first observation is that all three ablated variants keep ROUGE_L and BLEU very close to the full system, confirming that the front-end semantic parsing stage is already robust and that most modifications target the downstream SQL stage rather than the surface text. However, the SQL-related metrics react quite differently to different removals. Removing the structured patterns (Variant B) causes a clear drop in EM (0.84 → 0.63) and EX (0.91 → 0.67) even though ROUGE_L/BLEU remain high, which shows that text-level adequacy alone does not guarantee executable and schema-consistent SQL. Eliminating the hierarchical agent prompting (Variant C) leads to the largest degradation on SQL metrics (EM = 0.50, EX = 0.47) and also lowers CMA to 0.72, indicating that prompt engineering is the most sensitive component in the SQL synthesis stage. By contrast, removing the seven-domain constraint (Variant D) only mildly affects EM and EX (0.78 and 0.84, respectively) but yields the lowest CMA (0.77), suggesting that relaxing clinically required attributes mainly harms end-to-end patient matching on the 41-case HCC cohort rather than surface SQL accuracy. Overall, the ablation confirms that all three components contribute: structured patterns stabilize schema alignment, hierarchical prompting drives SQL correctness, and the seven-domain constraint ensures clinical completeness in real-world cohorts.

**Table 5:** Ablation study on the benchmark dataset and the real-world EHR cohort.

Variant	ROUGE_L	BLEU	EM	EX	CMA
(A) Full EC2Seq2Sql	**0.8067**	**0.8427**	**0.84**	**0.91**	**0.88**
(B) Without structured patterns	0.7810	0.8045	0.63	0.67	0.79
(C) Without agent prompting	0.8065	0.8420	0.50	0.47	0.72
(D) Without seven-domain constraint	0.8067	0.8427	0.78	0.84	0.77

### Real-world validation on hospital EHR data

To further assess the real-world applicability and clinical utility of the proposed EC2Seq2Sql framework beyond benchmark datasets, an independent validation experiment was conducted using de-identified EHR data obtained from the Clinical Research Unit of Zhongshan Hospital, Fudan University. This dataset consists of 41 patients diagnosed with HCC between 2021 and 2024. Each patient record contains structured and semi-structured fields relevant to clinical trial eligibility assessment, including age, gender, TNM stage, hepatitis B virus serological markers (HBsAg, HBcAb), history of biliary stones, surgical and chemotherapeutic history, targeted or immunotherapy administration (e.g., PD-1 inhibitors), CA19-9 biomarker levels, and histopathological findings. All data were fully anonymized in accordance with institutional ethics and data governance requirements to ensure compliance with privacy protection regulations. No personally identifiable information was accessed during data handling or model validation. During this validation, the structured EHR fields were aligned with the seven conceptual domains (condition, procedure, observation, laboratory, drug, age, and gender) used in EC2Seq2Sql, as detailed in the Supporting Information.

In this validation, eligibility criteria for advanced HCC clinical trials were selected from representative ClinicalTrials.gov protocols to emulate realistic recruitment scenarios. The BART-large-CNN model was employed to transform these free-text inclusion and exclusion criteria into structured semantic representations across seven conceptual fields—condition, procedure, observation, laboratory, drug, age, and gender. Subsequently, the LLM-based Agent component translated these structured representations into executable SQL queries using controlled prompt engineering, combining both system-level and expert-curated prompts. The generated SQL statements were executed directly on the hospital’s local EHR database, and the resulting patient subsets were independently reviewed by hepatology specialists to confirm their clinical validity.

Performance evaluation in this real-world setting was carried out using two complementary indicators:

(1) EX: the proportion of SQL statements successfully executed on the hospital EHR database without syntax or logical errors;

(2) CMA: the proportion of retrieved patient records independently verified by clinical experts as satisfying the corresponding inclusion and exclusion criteria. Formally, CMA is defined in Eq (7):

$$CMA=\frac{1}{N}\sum_{i=1}^{N}𝕀(P_{model}^{\left(i\right)}=P_{expert}^{\left(i\right)})$$ (7)

where $P_{model}^{\left(i\right)}$ and $P_{expert}^{\left(i\right)}$ denote the inclusion status determined by the model and by the expert review for the $i^{th}$ patient, respectively, and 𝕀(·) is the indicator function that equals 1 when both assessments agree.

All generated SQL statements were syntactically valid and executed successfully (EX = 1.00), demonstrating that the proposed framework can generate fully executable and logically consistent database queries within a real clinical data environment. The CMA reached 0.88, indicating that the vast majority of patients identified by EC2Seq2Sql were confirmed by hepatology experts to meet the eligibility requirements of the target clinical trial. These results confirm that the framework achieves not only technical correctness but also clinical relevance when applied to real-world hospital data.

Overall, the findings demonstrate that EC2Seq2Sql is capable of accurately translating natural-language eligibility criteria into executable and clinically valid SQL queries for EHR-based patient identification. This validation underscores the robustness, generalizability, and clinical utility of the framework, supporting its potential deployment in hospital informatics systems to facilitate efficient, reproducible, and scalable patient-trial matching workflows.

### Error analysis

To further elucidate the limitations of the EC2Seq2Sql framework and provide insights for future optimization, we conducted a qualitative error analysis on both the semantic parsing and SQL generation stages. A subset of samples from the test set was randomly selected for manual inspection. The model outputs were compared with the gold-standard references, and representative failure cases were categorized and analyzed to identify potential issues in structured conversion and logical reasoning. The common error types identified in this process are summarized in Table 6.

**Table 6:** Common error types and representative examples in the EC2Seq2Sql framework.

Stage	Error Type	Example	Effect
Semantic Parsing	Ambiguous terminology	recent surgery → procedure() (no time filter)	Missing temporal condition
Semantic Parsing	Nested logic loss	A and (B or C) → A and B	Simplified logical structure
Semantic Parsing	Over-generalization	hepatocellular carcinoma → liver disease	Reduced clinical specificity
SQL Generation	Field mapping mismatch	ALT → bilirubin column	Incorrect field linkage
SQL Generation	Negation inversion	no chemotherapy → chemotherapy=1	Reversed logical meaning
SQL Generation	Temporal operator error	within 6 months → > 6 months	Incorrect time constraint

**(1) Errors in the Semantic Parsing Stage.** In the semantic parsing process, a small number of generated structured representations showed inconsistencies or omissions compared with the reference annotations. The main error types are summarized as follows:

- *Ambiguous clinical terminology:* Expressions such as “recent surgery” or “active infection” were not sufficiently disambiguated, leading to structured outputs lacking temporal or status constraints.
- *Loss of nested logical relations:* Multi-layered logical structures (e.g., “A and (B or C)”) were sometimes flattened into a single layer, resulting in a loss of logical information.
- *Over-generalization:* The model occasionally replaced fine-grained medical terms with broader categories (e.g., “hepatocellular carcinoma” → “liver disease”), indicating limited ability to capture domain-specific granularity.

**(2) Errors in the SQL Generation Stage.** During SQL generation, some queries were syntactically valid but semantically inaccurate. The typical error types include:

- *Field mapping mismatch:* Certain medical entities were incorrectly mapped to EHR database fields, such as linking “ALT” to a bilirubin-related column.
- *Negation inversion:* Exclusion criteria containing negation (e.g., “no prior chemotherapy”) were occasionally misinterpreted as inclusion conditions, revealing ambiguity in the model’s handling of negation logic.
- *Temporal condition misplacement:* Temporal constraints (e.g., “within 6 months”) were sometimes converted using incorrect operators, leading to logic inversion.

These errors primarily arise from two sources: (i) the semantic gap between natural language and database logic representations, and (ii) the model’s limited contextual understanding of clinical text. To mitigate these issues, future work will focus on three directions: (a) integrating standardized medical ontologies (e.g., SNOMED-CT) to improve entity alignment and terminology normalization; (b) incorporating multi-hop reasoning mechanisms to better capture nested logical dependencies; and (c) refining prompt engineering strategies to explicitly encode negation and temporal logic. These enhancements are expected to improve both the semantic fidelity and execution robustness of the EC2Seq2Sql framework, thus providing a more reliable basis for clinical trial patient matching.

## Discussion

This study presents EC2Seq2Sql, an automated framework that converts narrative clinical trial eligibility criteria into executable SQL queries for patient screening. By combining a BART-based semantic parser with a GPT-4–driven agent, the system transforms complex free-text criteria into lightweight structured patterns and then into schema-grounded SQL over seven core domains (*condition, procedure, observation, laboratory, drug, age*, and *gender*) [7]. In doing so, it streamlines the traditional manual screening process and improves the transparency and reproducibility of cohort identification.

The experimental results show that introducing structured patterns improves text-level metrics, while the agent-based SQL generation achieves high EM, EX, and CMA, indicating both syntactic correctness and clinical usefulness of the generated queries. The seven-domain representation covers the majority of commonly used eligibility constraints, supporting application across diverse trial designs.

Meanwhile, recent cohort selection benchmarks suggest that LLMs can be promising for trial screening, while fine-grained clinical reasoning remains challenging [45]. Consistent with these observations, although EC2Seq2Sql demonstrates strong performance in clinical trial patient matching, several limitations remain. First, the BART model may struggle with ambiguous or highly context-dependent language, potentially leading to misinterpretation of logical operators or temporal qualifiers. Second, the agent may encounter difficulties in processing rare or highly specialized medical concepts, which can reduce the precision of the generated SQL for complex criteria. Third, restricting prompts to seven concept fields simplifies the design but may under-represent important factors such as prior treatment history, genetic markers, or detailed comorbidity profiles; as a result, some generated queries may omit clinically relevant constraints.

To address these limitations, future work will focus on the following directions:

**(1) Integration with EHR systems:** We plan to further integrate EC2Seq2Sql with hospital EHR platforms to build an end-to-end patient-matching service that can be invoked directly within clinical workflows. This includes developing robust interfaces to institutional databases, handling access control and logging, and evaluating the system prospectively in real recruitment scenarios.

**(2) Terminology standardization:** To further enhance cross-institutional interoperability and ensure robust medical concept interpretation, future work will integrate standardized medical ontologies such as SNOMED-CT and UMLS into the EC2Seq2Sql pipeline. Specifically, the structured outputs generated by the BART model will be mapped to standardized concept identifiers (e.g., SNOMED-CT concept IDs or UMLS CUIs) through a terminology normalization layer. This integration enables consistent representation of diseases, symptoms, procedures, and laboratory findings across heterogeneous EHR systems, thus reducing ambiguity and improving entity alignment. Additionally, ontology-based hierarchical relationships can support fine-grained reasoning (e.g., mapping “hepatocellular carcinoma” to its parent concept “liver cancer”), enhancing the precision and generalizability of patient matching in real-world settings. Such ontology-driven normalization has been proven effective in related systems such as Criteria2Query [18,20,21], and will be incorporated as a key component in future iterations of EC2Seq2Sql.

**(3) Scalability and adaptability:** To ensure the long-term applicability of EC2Seq2Sql across evolving clinical contexts, future work will focus on scalability and adaptability from three perspectives. (a) Handling evolving eligibility criteria: Clinical trial eligibility criteria evolve over time with advances in biomedical research and the introduction of new biomarkers and treatment modalities. To accommodate this dynamic nature, the EC2Seq2Sql framework can incorporate continual learning strategies that periodically retrain the BART semantic parser and the agent using updated datasets from ClinicalTrials.gov and institutional protocols. By leveraging incremental fine-tuning or adapter-based training, the model can adapt to newly emerging clinical terms and logic structures without catastrophic forgetting. Furthermore, ontology integration (e.g., SNOMED-CT, UMLS) provides a stable semantic backbone that supports longitudinal consistency when new entities or relations are introduced. (b) Multilingual and cross-lingual adaptation: Given the global nature of clinical trials, multilingual capability is essential. Recent advances in multilingual foundation models (e.g., mBART, XLM-R, GPT-4-turbo multilingual) can be incorporated to extend EC2Seq2Sql’s parsing capability to non-English eligibility criteria, such as Chinese or bilingual English–Chinese protocols [43,46]. Through translation alignment and shared multilingual embeddings, the framework can achieve cross-lingual semantic consistency, enabling eligibility parsing and SQL generation across diverse linguistic environments. (c) Multi-institutional and heterogeneous EHR integration: To enable deployment across hospitals with heterogeneous data schemas, the EC2Seq2Sql framework can be coupled with a schema mapping layer based on the OMOP CDM [18]. By normalizing local EHR structures to a unified schema, the system ensures consistent SQL generation and execution across institutions. Moreover, the modular agent design allows flexible integration with institution-specific APIs or databases, supporting federated or privacy-preserving query execution.

**(4) Practical implementation:** Finally, we aim to translate EC2Seq2Sql into deployable software modules that can be embedded into domestic clinical trial management systems, with user interfaces for clinicians and coordinators, logging and audit trails, and configuration options for different disease areas and institutions. This line of work will establish a robust technological foundation for broader clinical adoption.

In summary, these extensions will further enhance the scalability, interoperability, and real-world usability of the proposed framework, allowing it to evolve alongside changing clinical knowledge, diverse linguistic contexts, and heterogeneous institutional data infrastructures, thereby promoting broader applicability in real-world healthcare settings.

## Conclusions

This work addresses the gap between narrative clinical trial eligibility criteria and executable queries over real-world EHRs. We proposed EC2Seq2Sql, an end-to-end, two-stage framework that first parses free-text EC into lightweight structured patterns and then generates SQL through an agent-guided stage. The design is aligned with a seven-field EHR representation, making the approach implementable on typical hospital data.

In the benchmark experiments, the BART-based parsing module outperformed the compared baseline models on text-to-structure metrics, indicating that introducing structured patterns helps better preserve key clinical constraints. With the agent-based SQL generation, the system further achieved high exact-match and execution accuracy, indicating that the produced SQL is not only well-formed but also runnable. A supplementary test on 41 de-identified HCC cases from a real hospital cohort confirmed that the pipeline can be executed on real EHR data rather than only on public benchmark text.

Nonetheless, the current framework still assumes a controlled schema and single-center data, and performance may degrade for more diverse diseases or more complex criteria. Future work will focus on extending to multi-center and multi-disease settings, strengthening terminology normalization with SNOMED-CT/UMLS, and improving the robustness of prompt-based SQL generation so that the system can be deployed in heterogeneous clinical environments.

## Supporting information

S1 File Data mapping and real-world validation workflow. Supplementary Material A, including the data mapping schema (7-domain to EHR fields) and the real-world validation workflow used in Zhongshan Hospital EHR-based evaluation.
(PDF)
