# Supplementary material for: EC2Seq2Sql: Patient-trial matching with LLM agents
Source: PLoS One. 2026 Feb 12;21(2):e0341827. doi: 10.1371/journal.pone.0341827 (PMC12900307; doi:10.1371/journal.pone.0341827)
Supplement: S1 File — Supplementary Material A, including the data mapping schema (7-domain to EHR fields) and the real-world validation workflow used in Zhongshan Hospital EHR-based evaluation. (PDF) [file pone.0341827.s001.pdf]

## Data Mapping and Real-world Validation Workflow

### Supplementary Material A

### A.1 Data Mapping Schema

Each EHR record was aligned with the seven conceptual domains defined by EC2Seq2Sql, as summarized in Table 7.

**Table7 Mapping between conceptual domains and corresponding EHR fields in the real-world validation dataset.**

| Domain | Example Mapped EHR Fields |
|---|---|
| Condition | Primary diagnosis |
| Procedure | Surgical history, interventional therapy codes |
| Observation | Radiology and pathology reports |
| Laboratory | AFP, CA19-9, ALT, AST, bilirubin |
| Drug | Chemotherapy agents, targeted therapy, PD-1 inhibitor records |
| Age | Derived from date of birth and admission date |
| Gender | Male/Female indicator |

### A.2 Validation Workflow

(1) Extraction of anonymized patient records from Zhongshan Hospital's EHR system.
(2) Application of EC2Seq2Sql pipeline to generate structured representations and SQL queries.
(3) Execution of generated SQL queries within a controlled research database environment.
(4) Independent expert review of retrieved patient subsets to verify the correctness of inclusion/exclusion criteria.
(5) Calculation of EX and CMA according to the definitions in the main text.
